# Usage of do-not-attempt-to-resuscitate orders in a Swedish community hospital – patient involvement, documentation and compliance

**DOI:** 10.1186/s12910-020-00510-5

**Published:** 2020-08-01

**Authors:** Emilie Bertilsson, Birgitta Semark, Kristina Schildmeijer, Anders Bremer, Jörg Carlsson

**Affiliations:** 1 Department of Medicine, Section of Cardiology, Kalmar County Hospital, Kalmar, Sweden; 2 Faculty of Health and Life Sciences, Linnaeus University, Kalmar/Växjö, Sweden

**Keywords:** CPR, DNAR order, Informed consent, Ethics, Autonomy

## Abstract

**Background:**

To characterize patients dying in a community hospital with or without attempting cardiopulmonary resuscitation (CPR) and to describe patient involvement in, documentation of, and compliance with decisions on resuscitation (Do-not-attempt-to-resuscitate orders; DNAR).

**Methods:**

All patients who died in Kalmar County Hospital during January 1, 2016 until December 31, 2016 were included. All information from the patients’ electronic chart was analysed.

**Results:**

Of 660 patients (mean age 77.7 ± 12.1 years; range 21–101; median 79; 321 (48.6%) female), 30 (4.5%) were pronounced dead in the emergency department after out-of-hospital CPR. Of the remaining 630 patients a DNAR order had been documented in 558 patients (88.6%). Seventy had no DNAR order and 2 an explicit order to do CPR. In 43 of these 70 patients CPR was unsuccessfully attempted while the remaining 27 patients died without attempting CPR. In 2 of 558 (0.36%) patients CPR was attempted despite a DNAR order in place. In 412 patients (73.8%) the DNAR order had not been discussed with neither patient nor family/friends. Moreover, in 75 cases (13.4%) neither patient nor family/friends were even informed about the decision on code status.

**Conclusions:**

In general, a large percentage of patients in our study had a DNAR order in place (88.6%). However, 27 patients (4.3%) died without CPR attempt or DNAR order. DNAR orders had not been discussed with the patient/surrogate in almost three fourths of the patients. Further work has to be done to elucidate the barriers to discussions of CPR decisions with the patient.

## Background

A large percentage of the population in western countries are dying in hospitals and institutions instead of in their own homes. In Sweden about 40% of all deaths in 2016 occurred in the hospital and almost 40% in institutions for the elderly [1]. Cardiopulmonary resuscitation (CPR) has become a default treatment despite the fact that it was initially not intended to hinder patients from dying a natural death [2]. It seems that CPR is the only medical treatment for which the patients consent is sought for not receiving it [3]. The fact that many in-hospital patients have irreversible and even terminal conditions but could be subjected to default CPR with all its’ possible harms has led to the development of do-not-attempt-resuscitation (DNAR) orders. The decision making process, the understanding and communication of DNAR orders has repeatedly being reported to be hampered by misunderstandings, possible discrimination issues, cultural differences and inconsistencies [4–15]. Furthermore, the decision more often than not is based on concepts like futility and ideas of a life worth living, −both of which are highly subjective [16–20]. Therefore, and in order to protect patient autonomy, it is mandatory to include the patient and, when appropriate, family members in the decision making concerning CPR. However, the legal requirements around DNAR orders and the practice vary considerably across countries [6, 7, 13]. In Sweden a patient cannot demand CPR if the physician judges such treatment not being “for the benefit” of the patient [21, 22]. However, the DNAR decision should be discussed with the patient or a representative and documented in the patient chart [21, 22].

We aimed at characterizing patients dying in a community hospital with or without attempting CPR excluding those from further analysis who were pronounced dead at the hospital after out-of-hospital resuscitation [23]. The main interest of the study was patient involvement in, documentation of, and compliance with valid DNAR orders. Other areas of interest were the documented reasons for and wording of DNAR orders and whether the patients died alone or in the presence of family members or hospital personnel.

## Methods

All patients who died in or were pronounced dead in Kalmar County Hospital during January 1, 2016 until December 31, 2016 were included in this retrospective study. All pertinent information from the patients’ electronic chart was analysed by a cardiology nurse (E.B.) together with a senior cardiologist and medical ethicist (J.C.). Specifically, it was noted whether a DNAR order was highlighted on the first page of the electronic patient chart (there is a symbol on the left upper side, directly beside the patient’s name and personal number indicating important information such as occurrence of a DNAR code, allergies, anticoagulation, presence of an implanted device and previous statements of the patient concerning certain treatments, e.g. blood transfusions). It was then checked whether there was a corresponding text in the patient’s chart, written by the physician in charge, concerning the reasons for the DNAR order, whether or not it had been discussed with the patient, what kind of information the patient had received during the consultation, whether anyone else had been present and whether the patient had forwarded own thoughts about the issue of CPR and whether these were in accordance with the physician’s recommendation. We defined informing as giving information to the patient about the disease and the likelihood of CPR outcome while discussing was defined as inquiring into the thoughts and expectations the patient has concerning a possible CPR situation. Doubtful cases were discussed with the other authors (B.S., K.S., A.B.). The study was approved by the Regional Ethic review Board in Linköping, Sweden; 2017/270–31.

## Results

Of 660 patients (mean age 77.7 ± 12.1 years; range 21–101; median 79; 321 (48.6%) female), 30 (4.5%) were pronounced dead in the emergency department after out of hospital CPR (OHCPR). These patients were significantly younger (69.5 ± 15.0 versus 78.1 ± 11.7 years; *p* < 0.01) and less often female (26.7% versus 49.7%; *p* = 0.01) than those who died in the hospital. Twenty-five (83.3%) patients had no statement concerning CPR in their patient chart, 4 had a DNAR order from a previous hospital stay and one had a do-CPR statement. These data were significantly different from in-hospital patients were 558 of 630 (88.6%) did have a DNAR order. The OHCPR patients were excluded as planned from further analysis.

The place of death of the 630 patients was as follows: 11 (1.7%) in the emergency department (CPR had first been started in the ER after cardiac arrest in the ER), 536 (85.1%) on the ward, 74 (11.7%) in the intensive care unit, 6 (1.0%) in the radiology department and 3 (0.5%) in the operating room. DNAR orders were in place in 43 (58.1%) of the 74 patients who died in the intensive care unit compared to 509 (95%) of the 536 patients who died on the ward.

CPR had been attempted in 47 of the 630 (7.5%) patients who died in the hospital, CPR was performed for 17.9 ± 14.2 min (range 1–68 min). Two of these patients had a DNAR order in place (CPR duration 25 and 40 min), another two had a do CPR statement documented in their chart (CPR duration 10 and 25 min) and the remaining 43 had no order (CPR duration 17.3 ± 14.4 min, range 1–68). During the same time, 19 patients survived in-hospital CPR, none of whom had a DNAR order. In 583 of the 630 (92.5%) patients who died in the hospital without CPR attempt, 556 had a DNAR order documented in their chart while 27 did not have any order in place. Therewith, a total of 29 patients were not treated according to standards of care with 27 not receiving CPR despite no DNAR order in place and 2 receiving CPR despite a DNAR order in the chart. The reason for CPR despite DNAR was lack of information about patients’ code status and not a conscious decision to overrule a valid decision.

The underlying diseases are listed in Table 1 according to the coding of the physician in charge of the patient. Individual patients could have a coding of one or more diseases or even multimorbidity when two or more chronic conditions were thought to be more important that separate diagnoses.

**Table 1:** Underlying diseases according to physicians’ coding of main diagnoses

Disease	N
Heart disease	311
Cancer	186
Multimorbidity	139
Diabetes mellitus	107
Lung disease	96
Stroke	64
Neurologic disease	55
Dementia	55
Psychiatric disease	52
Kidney failure	50
Autoimmune and endocrine disease	42
Gastrointestinal disease	39
None	23
Hematologic disease	10
Unknown	3

The 27 patients who had neither a DNAR order nor had received CPR directly before they died were 67.5 ± 16.7 years old (range 25–95), and 13 (48.1%) were female. Nine (33.3%) patients had survived CPR with a duration of 43 ± 28 min (range 2–80) before they finally died. Nine patients were considered brain dead and one had been unconscious for 169 h before death. In 12 cases (44.4%) relatives were informed about the “critical status” or the “gloomy prognosis” of the patient. In one case the word “death” had been used. The strategy of “allow natural death” was not explicitly mentioned in the chart although it was apparently used in these patients.

The time between DNAR documentation and death was between 0 and 2645 days (mean 26.4; median 4). Documentation had been done on admission in 91 cases (16.3%), during hospital stay in 311 cases (55.7%) or was from an earlier hospital stay in 81 patients (14.5%). An earlier order was confirmed on admission in 40 cases (7.2%) while confirmation of an earlier order was delayed until later during hospital stay in 35 cases (6.3%).

In 412 patients (73.8%) the DNAR order had not been discussed with either patient or family members (Table 2). Moreover, in 75 cases (13.4%) neither patient nor family/friends were even informed about the decision on code status (Table 2). These 75 cases were analysed concerning possible reasons for not informing patient, family or friends. In 40 (53.3%) the patient was either unconscious (*n* = 20) or judged not to be able to understand information about CPR (n = 20). Of the 40 cases where patient information was judged to be impossible, 38 patients had a family which wasn’t informed either. It was not possible to retrospectively sort out the reasons for not informing the family. In the remaining 35 cases there was no information at all in the chart concerning the reasons the patient wasn’t informed about the DNAR order.

**Table 2:** DNAR orders and documentation of discussion and information. The order had been discussed in 13.6% with the patient, in 12.6% with family and in the remaining 73.8% with neither patient nor family

	n	Age at death* [years] (range)	DNAR order present [n; %]	Time between DNAR and death [days]	Discussion about DNAR order with patient [n; %]	No information given to patient/relatives about DNAR order [n;%]
All patients	630	78.1 ± 11.8 (21–101)	558 (88.6%)	26.4 ± 145 (0–2645)	76 (13.6%)	75 (13.4%)
Female	313	79.4 ± 11.6 (25–101)	283 (90.4%)	28.9 ± 168 (0–2645)	39 (13.8%)	35 (12.4%)
Male	317	76.7 ± 11.8 (21–96)	275 (86.8%)	23.9 ± 118 (0–1568)	37 (13.5%)	40 (14.5%)

Female patients were significantly older at the time of death than male patients (* *p* < 0.01)

Swedish health law stipulates documentation of the reasons for DNAR orders and consultation of other health care professionals concerning the order which had been done in 352 (63.1%) cases [22]. Swedish ethical guidelines suggest that “No CPR” should be used to document ab DNAR order. However, the term “0 CPR” was used in 279 (50%) of the charts while “No CPR” was used in 252 (45%) cases. In the remaining 27 (5%) charts were other wordings used [21]. Furthermore, the DNAR order, which is highlighted and easily visible on the first page of the electronic chart, should be accompanied by an explanatory text written at the same occasion. Compliance with that requirement was 59.5% (332/558). The explanatory text stated most commonly high age (72 cases, sometimes expressed as “biological age”), metastasized cancer (91 cases), comorbidity (70 cases), and dementia (35 cases). Some other explanations were alcoholism and anatomy that makes CPR impossible.

The cause of death was documented as sudden cardiac arrest in 67 cases, respiratory disease in 65 cases, metastasized cancer in 112 cases, heart failure in 42 cases, multiorgan failure in 42 cases, infection (including pneumonia) in 61 cases and sepsis in 25 cases. The remaining 246 cases had other causes of death or combinations of mentioned causes.

The patient died more often in the presence of a relative (273/558; 48.9%) if there was a DNAR order in place than if there was no DNAR order (7/70; 10%). Health care personnel was present in 178/558 (31.9%) in cases with DNAR order and in 63/70 (90%) without DNAR order. None was present in 119/558 (21.3%) with DNAR order and 3/70 (4.3%) without DNAR order. In 56/558 (10%) with DNAR order and 2/70 (2.9%) without DNAR order there was no documentation about other people present at death. Please note that the numbers do not correspond to 100% because of possible presence of a relative as well as health care personnel.

## Discussion

The latest European guidelines for resuscitation state that large differences exist between European countries regarding the practice and attitudes towards CPR [24]. This is based on differences in the cultural, ethical, in part religious and juridical context. CPR has become almost a default treatment despite that it originally was described for reversible causes of cardiac arrest in the hospital [2]. In order to avoid CPR attempts that are deemed to be medically futile or not in the best interest of the patient, DNAR orders have been introduced [3, 8]. The practice and attitudes towards these orders vary substantially between countries and hospitals [4–15, 25]. In our population of patients dying in a community hospital, a large percentage of patients had a DNAR order in place (88.6%) and compliance with the order was high (99.6%) with only two patients with a DNAR order receiving CPR. However, 27 patients (4.3%) died without CPR attempt or DNAR order. Analysis of these patients was hampered by lack of explicit documentation but death may have been accepted in these cases, but this can only be inferred from knowledge of the cultural rather than a chart review. These patients were, if anything should be said about differences, even sicker than the majority of patients with a DNAR order and 19 (70%) of these patients died in the intensive care unit. Whether or not withholding CPR in these cases should be regarded as an ethical violation deserves further investigation into the reasons for this practice which is not in accordance to guidelines.

The occurrence of DNAR orders in Sweden seems to be stable over time as Aune et al. reported 82% already in 1999 from Gothenburg [25]. Although they did not clearly state their applied criteria, they concluded that there was no case where withholding CPR had been unethical. However, the definition of ethical justifiability of withholding CPR or defining a time limit for CPR [19] is clearly a problem. The absence of a universally agreed upon definition of futility [20] might be used to motivate CPR in virtually all cases but those where the patient actively decides against it. On the other hand, vague and not verbalized concepts of futility hold and applied by individual physicians are not suitable to facilitate patients’ own decision making. Ethical justifiability of CPR decisions therefore requires information of the patient and incorporation of patients’ ideas and expectations into the decision making. This point might best be illustrated by an individual case: a female patient with metastasized lung cancer and chemotherapy-induced cardiomyopathy suffers from ventricular fibrillation. Whether or not an instant death in ventricular fibrillation is preferable to a death because of cancer in a couple of weeks is clearly not for the physician to decide. The value of the remaining weeks of life cannot be judged by anybody else but the patient. This expression of autonomy has to be guarded by health care personnel through informing and discussing the issue of CPR with the patient. And while guidelines therefore require discussion of DNAR orders with the patient and/or family members, this had not been done in almost three fourths of the patients of this study. The reasons for this cannot be concluded from the present study which was only able to analyse accessible documentation. Thoughts and clinical judgements that were not documented could not be assessed. This would require an interview study with the involved health care personnel. A recent paper from Sweden investigating the attitudes of nurses and physicians towards DNAR orders reported that the reluctance of physicians to discuss end of life issues with the patient was often based on non-maleficence: the information of the patient on CPR code status might cause stress and harm [4].

Furthermore, the compliance with Swedish health care law was low concerning the form of the DNAR orders. In 26% of the cases the order was not accompanied by an explanatory text. In addition, ethical guidelines were not being followed in many cases where the accompanying text contained only “dementia” or “age” as explanation for the DNAR order without further comment. Increasing age has been identified as being connected to the usage DNAR orders [5]. However, whether this represents discrimination or “ageism” remains unclear even in our investigation where the patients with a DNAR order were older than those without an order (78.8 ± 11.4 versus 72.2 ± 13.3 years; *p* < 0.01). For one, older people were in general sicker than younger and secondly, we did not investigate the general usage of DNAR orders in all hospitalized patients. Therefore, the major limitation of this investigation is that we have only analysed DNAR orders in connection to patients who died in the hospital. The overall use of DNAR orders in our hospital is currently unknown as many patients are discharged from the hospital alive with an order in place. In our study, 81 patients (14.5%) who died during 2016 had had a DNAR order from a previous hospital stay. The literature about the usage of DNAR orders in hospital populations is sparse. A small study from the UK reported occurrence of these orders in 28.4% of medical inpatients [15]. Another limitation is that some discussions with patients and their family and friends might have taken place without documentation. However, even if this might in some cases be ethically acceptable, it is not according to the law and might need to unnecessary treatment and confusion of health care personnel not aware of these undocumented decisions. Obviously, even if the discussion and information is documented, little is known about patients´ understanding of the implications of CPR [26].

## Conclusions

In general, a large percentage of patients in our study had a DNAR order in place (88.6%). However, 27 patients (4.3%) died without CPR attempt or DNAR order. DNAR orders had not been discussed with the patient/surrogate in almost three fourths of the patients. Further work has to be done to investigate the barriers for medical personnel to discuss the issues and implications of CPR and DNAR orders with the patient.

## Data Availability

The datasets used and analysed during the current study are available from the corresponding author on reasonable request.

## Abbreviations

CPR: Cardiopulmonary resuscitation
DNAR order: Do-not-attempt-resuscitation order
